# Temporal Dynamics of Distinct CA1 Cell Populations during Unconscious State Induced by Ketamine

**DOI:** 10.1371/journal.pone.0015209

**Published:** 2010-12-08

**Authors:** Hui Kuang, Longnian Lin, Joe Z. Tsien

**Affiliations:** 1 Key Laboratories of MOE and STCSM, Shanghai Institute of Brain Functional Genomics, East China Normal University, Shanghai, China; 2 School of Medicine, Brain and Behavior Discovery Institute, Georgia Health Sciences University, Augusta, Georgia, United States of America; INSERM U901, France

## Abstract

Ketamine is a widely used dissociative anesthetic which can induce some psychotic-like symptoms and memory deficits in some patients during the post-operative period. To understand its effects on neural population dynamics in the brain, we employed large-scale *in vivo* ensemble recording techniques to monitor the activity patterns of simultaneously recorded hippocampal CA1 pyramidal cells and various interneurons during several conscious and unconscious states such as awake rest, running, slow wave sleep, and ketamine-induced anesthesia. Our analyses reveal that ketamine induces distinct oscillatory dynamics not only in pyramidal cells but also in at least seven different types of CA1 interneurons including putative basket cells, chandelier cells, bistratified cells, and O-LM cells. These emergent unique oscillatory dynamics may very well reflect the intrinsic temporal relationships within the CA1 circuit. It is conceivable that systematic characterization of network dynamics may eventually lead to better understanding of how ketamine induces unconsciousness and consequently alters the conscious mind.

## Introduction

Ketamine is a phencyclidine derivative described in 1965 [1], and was introduced into clinical practice by 1970 as an agent for general anesthesia [2]. Ketamine is quite a unique anesthetic drug because it demonstrates analgesic, hypnotic and amnesic effects [3], [4]. It induces an anesthetic state referred to as “dissociative anesthesia”, in which the patient often has his or her eyes open yet feels disconnected from the environment [1]. The patient is unconscious, amnesic and deeply analgesic under higher dosages of ketamine. In other words, the patient is incapable of associating the input of afferent stimuli and the integration of information and signals to the conscious mind is reduced or blocked [4]. Dissociative anesthesia produced by ketamine has been postulated to be a result of reduced activation in the thalamocortical structures and increased activity in the limbic system and hippocampus [1]. Moreover, after waking from ketamine-induced general anesthesia, about 5∼30% of patients report psychotic-like symptoms including delirium and hallucinations [5]. In addition, the incidence of illicit, recreational abuse of ketamine has increased in the past decades, which produces general decreased awareness of the environment, sedation, vivid dreams, increased distractibility, disorientation, and feelings of invulnerability. In some cases, it can lead to intense hallucinations, impaired thought processes, out-of-body experiences, and changes in perception about body, surroundings, time, and sounds.

Ketamine acts primarily as an uncompetitive NMDA receptor antagonist, and has been used to probe its effects on memory processes [6]–[8]. Its amnesic effects are thought to be, in part, due to its action on the hippocampus [9]–[12]. The hippocampus belongs to the limbic system and is known to be crucial for memory processes [13]–[22]. Despite the wide use of ketamine in general anesthesia and memory research, characterization of the ketamine's effects on hippocampal cell populations has been mostly limited to *in vitro* brain slices or using EEG methods [23]–[26]. Little is known about its detailed action on dynamic patterns of hippocampal cells *in vivo*. Moreover, since the composition of the NMDA receptors differs significantly among different types of neurons in the hippocampus [27]–[30], ketamine may produce distinct changes on various types of hippocampal pyramidal cells and interneurons. For example, NR2A and NR2B mRNA are expressed mainly in pyramidal cells, whereas NR2C and NR2D mRNA are present in different subsets of hippocampal interneurons [27]–[30]. In addition, ketamine can act on other receptors or channels including the GABAa receptor, nicotinic and muscarinc Ach receptors, and opioid receptors, etc [3], [31] which also vary among different cell types. Thus, *in vivo* effects of ketamine on the neural populations can be diverse and complex.

Given the fact that excitatory neurons and interneurons work together to produce cooperative network properties [32]–[36], it would be highly useful to study the *in vivo* effects of ketamine on both pyramidal cells and interneurons and their possible interactions in the simultaneously recorded population. In the present study, we employed an *in vivo* ensemble recording technique and simultaneously recorded large numbers of CA1 cells in the mouse hippocampal CA1 region in response to ketamine injections. In consideration of ketamine's hypnotic effect, we further compared ketamine-induced CA1 dynamic changes with those observed during sleep as well as during awake resting and running states. These experiments have allowed us to determine various CA1 cells' dynamics and characteristics associated with the wakeful states and two unconscious states.

## Results

### Ensemble *in vivo* recording from hippocampal CA1

**Figure 1:**
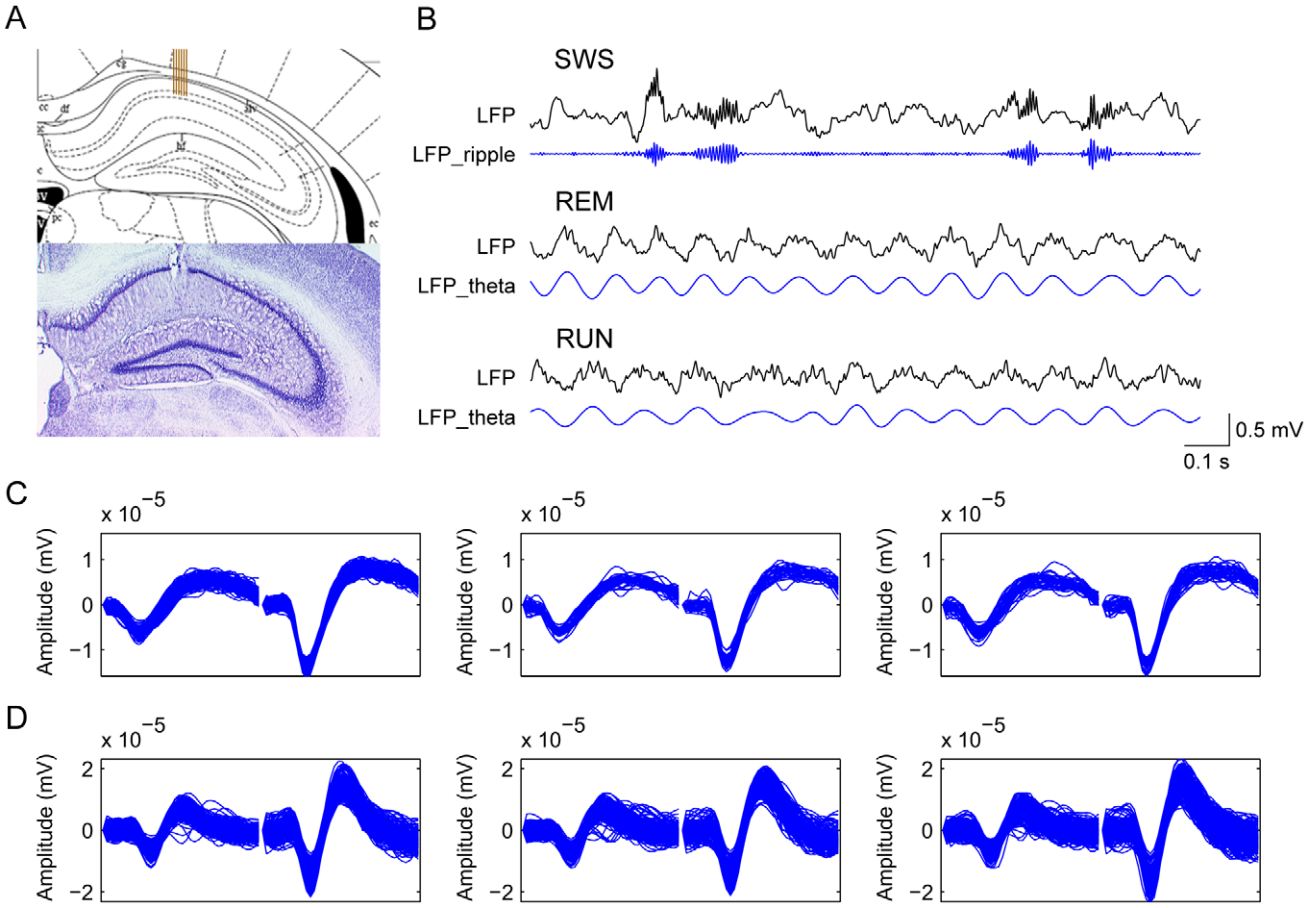
Ensemble *in vivo* recording in the CA1 region of the mouse hippocampus. (**A**) Histological confirmation of the electrodes position. The top panel shows the atlas of the mouse brain, the brown bars illustrate the position of electrode arrays with the tips in the pyramidal cell layer. The lower panel shows Nissl staining in a hippocampal slice; the small holes indicating the actual position of the electrode bundle marked by a small amount of current. (**B**) The characteristic oscillations also confirm the recording happened in the hippocampal CA1 region. The top panel shows an example of LFP recorded from one channel during SWS, and the filtered LFP shows high-frequency ripple (100–250 Hz). The middle and lower panels show the LFP recorded when the animal was in REM and running, and LFP_theta indicates the characteristic theta oscillations (4–12 Hz). Vertical scale bar represents 500 mV for LFP, horizontal scale bar represents 0.1 sec time. (**C**, **D**) Stable recordings were confirmed as judged by the waveforms of recorded cells at the beginning (left subpanel), during (middle), and end (right) of the experiments. One representative putative pyramidal cell (**C**) and one representative putative interneuron (**D**) are shown here. The waveforms were plotted from a 100-sec recording in the beginning (upper row) and end (lower row) of recordings.

Using large-scale *in vivo* neural recording techniques that we have recently developed [37]–[39], we simultaneously recorded the spike activity of multiple neurons as well as local field potentials from the CA1 region of the mouse hippocampus. We confirmed that our recordings were taken place in the CA1 region of hippocampus by post-experiment histological staining of the electrode positions (Figure 1A). During the experiments, we also used two *in vivo* criteria for assessing the electrodes' positions in CA1: (1) large amplitude high frequency ripples (100–250 Hz) during slow-wave sleep (SWS) (Figure 1B) [37], [40]–[44]; (2) characteristic theta rhythm oscillations (4–12 Hz) during rapid eye movement sleep (REM) or running [41]–[44] (Figure 1B). These characteristic physiological markers, together with histological staining data, ensure the datasets collected were from the CA1 region of the mouse hippocampus.

Principal excitatory units (putative pyramidal cells) and inhibitory units (putative interneurons) were discriminated based on spike duration, firing rate, and autocorrelogram [37], [42]–[44] [45](see Materials and Methods for details). Pyramidal cells constituted the majority of the recorded cells in the CA1 region. The stable recordings were confirmed by comparing the spike waveforms of the unit at the beginning, duration, and end of the experiment (Figure 1C for putative pyramidal cells, and 1D for putative interneurons). A total of 310 well separated, stable, and simultaneously recorded units from four mice were used for the detailed analyses.

We calculated the neuron firing rates by using 300 seconds of data recorded during the awake resting state before ketamine injection and during the unconscious state which usually occurred within several minutes after the ketamine injection (see Table 1). It should be noted that after a single dosage of ketamine (100 mg/kg of body weight, i.p.), the onset of anesthetic effects, as measured by the loss-of-righting-reflex assay, was observed within three minutes, and recovery typically began after 20 minutes to 1 hour. We observed that on average, the firing rate of putative pyramidal cells significantly decreased after ketamine injection (see Table 1). For interneurons, the average firing rate after ketamine injection was slightly greater than that during sleeping and awake rest states, but slightly smaller than that during running (see Table 1). This suggests that the excitatory neural activity of the hippocampus was suppressed while the inhibitory neural activity was not changed much when the mice were sedated by ketamine injection. This is quite interesting because it has been long postulated that ketamine might increase activity in the limbic system and hippocampus [1].

**Table 1:** Spike duration and firing rates of different CA1 neuron classes.

	N	Spike duration (usec)	Averaged firing rate (Hz)				
			Awake rest	Run	Sleep	Ketamine	p value
**Pyr**	211	354.90±3.03	1.33±0.12	1.95±0.15 *	1.55±0.09 *	1.08±0.11	4.63E-06
**Int**	99	205.32±4.69	11.18±0.88	14.92±1.15	8.60±0.71	11.91±0.97	0.0001
**Type 1**	19	217.04±8.32	13.12±2.42	19.19±3.15	11.38±2.02	11.65±2.23	0.1018
**Type 2**	22	229.75±7.72	6.40±1.04	12.15±2.57	9.01±1.25	6.97±1.61	0.0851
**Type 3**	1	167.25±0.00	15.33±0.00	17.54±0.00	12.89±0.00	10.34±0.00	NaN
**Type 4**	2	220.63±26.38	25.36±8.83	27.00±4.71	28.57±13.83	14.53±12.79	0.7891
**Type 5**	11	227.39±19.99	9.13±2.12	11.13±2.62	5.82±1.36 *	13.41±1.20	0.0507
**Type 6**	15	168.75±7.50	19.91±1.74	22.78±2.06	8.22±1.00 *	21.43±2.47	2.46E-06
**Type 7**	7	155.82±6.08	14.98±1.91	19.30±3.54	9.99±2.16	14.98±3.06	0.1542
**Other**	22	200.74±9.61	6.68±1.32	7.95±1.53	4.98±0.91	8.69±2.02	0.3249

The averaged spike duration and firing rates of recorded putative pyramidal cells and putative interneurons are summed in the above table. And the averaged spike duration and firing rates of different subclasses of interneurons are list behind. The averaged firing rates were calculated using 300 sec of data during each state (awake rest, running, sleep and ketamine-induced anesthesia). One-way ANOVA with a post-hoc test was performed to detect the significance between different states. (*p<0.05 compared with the value under ketamine).

### Synchronous firing of pyramidal cells induced by ketamine

**Figure 2:**
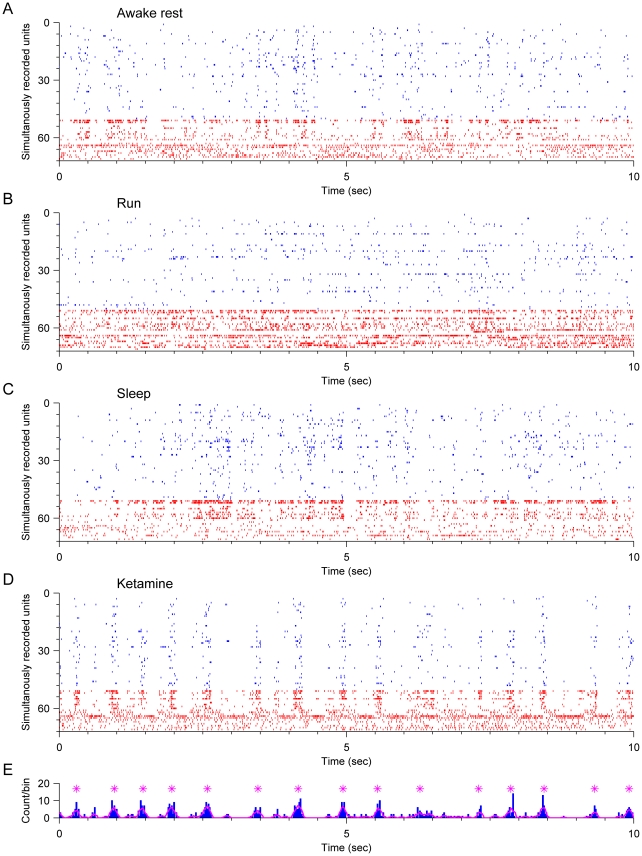
Hippocampal ensemble firing pattern changes after ketamine injection. (**A–E**) The spike raster plots show the activities of simultaneously recorded hippocampal neurons when the animal was asleep (A), awake resting (B), running (C), anesthetic state after ketamine injection (D), and recovery from ketamine (E). Each short vertical bar indicates a single spike, and each line indicates a single unit recorded. Pyramidal cells are shown in blue, and interneurons are shown in red. After ketamine injection, the population firing pattern has changed dramatically. Note the vertical alignments of spikes indicating the synchronous firing of pyramidal cells (D).

In contrast to the small, but significant, changes in average firing rates of putative pyramidal cells, the major effect of ketamine on CA1 was the dramatic change in ensemble dynamic patterns. Without ketamine, pyramidal cells fired at low frequencies and in a desynchronized fashion (Figure 2A–2C for sleep, awake rest and running, respectively). Interestingly, after ketamine injection, the pyramidal cells began to exhibit a great level of synchronous firing. This is evident from the rhythmic alignment of firing spikes of pyramidal cells in the spike raster plot (Figure 2D). As the animal started recovering from ketamine, the unique synchronized firing pattern began to fade away (Figure 2E). It is observed that the synchrony epochs occurrence became smaller and less regular than that during the effective anesthesia stage induced by ketamine.

**Figure 3:**
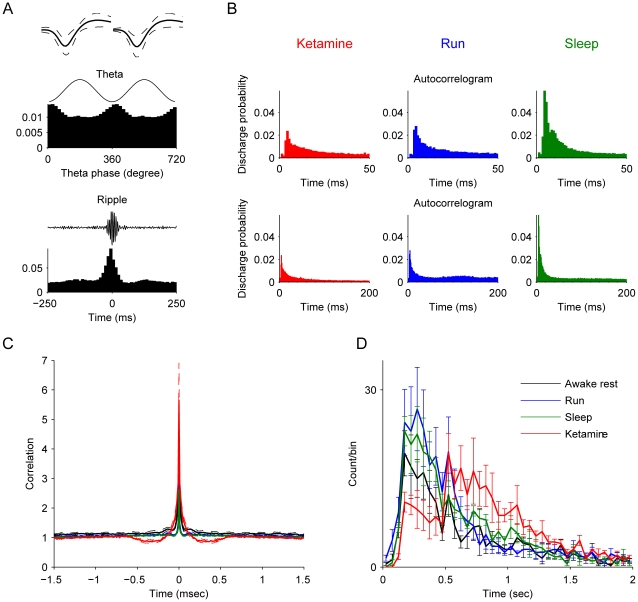
Pyramidal cell properties in the absence or presence of ketamine. (**A**) Averaged spike waveforms of 211 recorded pyramidal cells by using stereotrode are shown in left upper panel, solid lines represent the mean spike waveforms and the dashed lines indicate the mean±S.D. The middle and lower left panels illustrate the spike timing of pyramidal cells with relationship to theta oscillations and ripple epochs respectively. (**B**) The average auto-correlations of pyramidal cells 5 min after ketamine injection, and during sleep and running are shown. Autocorrelograms are plotted in two different time scales. (**C**) Auto-correlation function of joint-activities of pyramidal cells. (**D**) Averaged interval histograms of pyramidal cell synchronous epochs over animals. Note the peak about 0.6 sec of the red line indicates that pyramidal cells exhibited slow rhythmical firings.

To characterize the effect of ketamine on the firing patterns of hippocampal pyramidal cells, we examined the firing properties of pyramidal cells (Figure 3A) and the auto-correlation function for spikes before and after ketamine injection (Figure 3B). As expected, the auto-correlation of putative pyramidal cells during the sleep and running states exhibited a typical sharp peak between 3–5 ms (Figure 3B and C, green and blue subplots), which indicates pyramidal cells firing occasional complex-spike bursts. After ketamine injection, this prominent peak remained, but was smaller than that of before (Figure 3B and C, red subplot), which may reflect the significant decrease of firing rates. Then we reconstructed the joint-activity data pooled from the activities of simultaneously recorded pyramidal cells, and calculated the auto-correlation functions. We also binned and smoothed the joint-activities, then filtered the data with 1 S.D. above mean, then the peaks were found to use as the pyramidal cells synchronous firing epochs. Most significantly, the auto-correlation function of the joint-activities of pyramidal cells, as well as the interval histogram of synchronous firing epochs, confirmed that the pyramidal cell population exhibited rhythmical firings with an average frequency of 1.47 Hz (as indicated by the second peak in Figure 3C and D). The average low frequency oscillation across all recorded mice was 1.4±0.1 Hz.

### Diverse responses of interneurons upon ketamine injection

While the pyramidal cell population exhibited uniform synchronization in response to ketamine, GABAergic interneurons had much more diverse patterns, which may reflect a wide range of distinct types of interneurons [32]–[34], [46]. After ketamine injection, interneurons showed varied change in firing frequencies and firing patterns (Figure 2D, spikes in red color), some units fired synchronously with pyramidal cells, a few units had long bursts of over hundreds of milliseconds with a regular rhythm (about 1–2 Hz), and some other interneurons did not exhibited change.

To further define these interneurons, we classified them not only by the action potential waveforms and the timing of the spike activities of inhibitory units during theta oscillations and sharp-wave associate ripples, but also by the unique dynamic change induced by ketamine. We plotted autocorrelograms during different states, in relationship with slow-wave sleep (SWS), running (RUN), and the unconscious state induced by ketamine injection (KET) for each interneuron, as well as the cross-correlation function with the joint-activity of simultaneously recorded pyramidal cells. According to these characteristics, we classified recorded interneurons into seven main subclasses.

**Figure 4:**
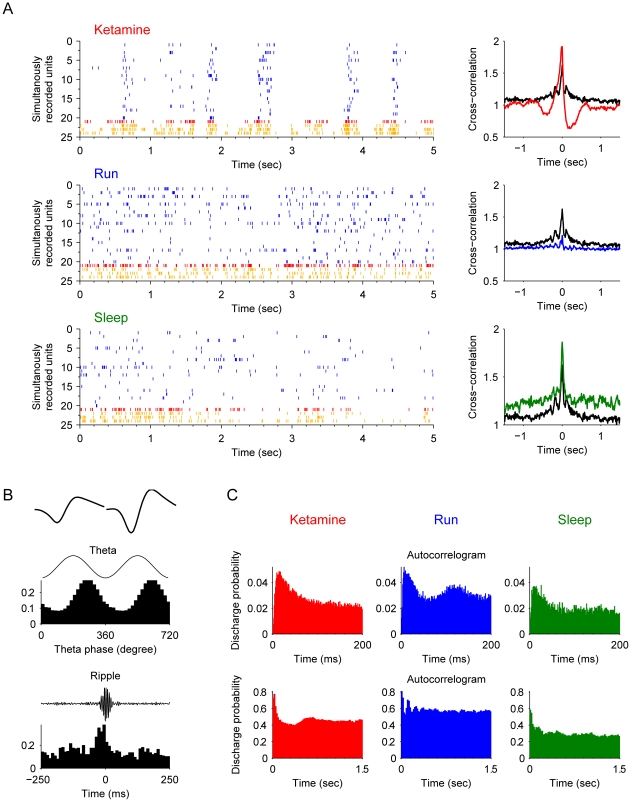
Properties of type-1 interneuron (putative *basket cells*). (**A**) Spike rasters of silmultanously recorded pyramidal cells (blue) and basket cells (example plotted here in red, remaining units in orange) after ketamine injection, during running and slow-wave sleep are shown (left panels). The cross-correlation functions of the interneuron and pyramidal cell joint-activities are plotted on the right. The black line is the cross-correlation during the awake rest state. (**B**) The basic properties of this example basket cell. The most upper left panel shows the average spike waveforms of this unit. The middle left panel illustrates the strongly coupled spike timing of this unit with theta oscillations (p<0.001, Rayleigth's test). The lower left panel shows the significantly elevated firing rate during ripple epochs. (**C**) Autocorrelograms in different states: after ketamine injection, during running, and during sleep respectively. Autocorrelograms are shown in two time scales for better illustration: 0–200 ms and 0–1.5 sec.

Type-1 interneurons have characteristic firing properties belong to the putative *basket cells*. These basket cells were highly synchronized with the pyramidal cells after ketamine injection (Figure 4A). These units were usually obtained simultaneously with putative pyramidal cells, indicating their proximal location to pyramidal cell bodies. They had uniform asymmetrical spike waveforms (Figure 4B, upper subpanel) and fired in phase with theta oscillations (Figure 4B middle subpanel). Third, they exhibited elevated firing during ripple epochs (Figure 4B, lower subpanel). During sharp wave-associated ripple episodes, these basket cells increased their firing frequency at the highest amplitude of the ripple (Figure 4B). The overall autocorrelograms were similar during the ketamine (KET), running (RUN), or slow-wave sleep states (SWS) (Figure 4C, subpanel in top row). However, autocorrelograms of these basket cells recorded during running revealed a second small peak about 120–130 ms during running (Figure 4C, the second subplot in the upper row). This fits well with the fact that these basket cells fire at the descending phase of the theta oscillations (8–9 Hz) during running (see the second peak in the top blue subplot of Figure 4C). After ketamine injection, this second peak disappeared (the upper red subplot of Figure 4C), which remained consistent with the diminishing of theta oscillations in local field potentials. However, another small peak appeared at about 0.65 sec (the lower red subplot of Figure 4C), consistent with the oscillations at this low frequency (1.4 Hz) coupled with the pyramidal cells.

**Figure 5:**
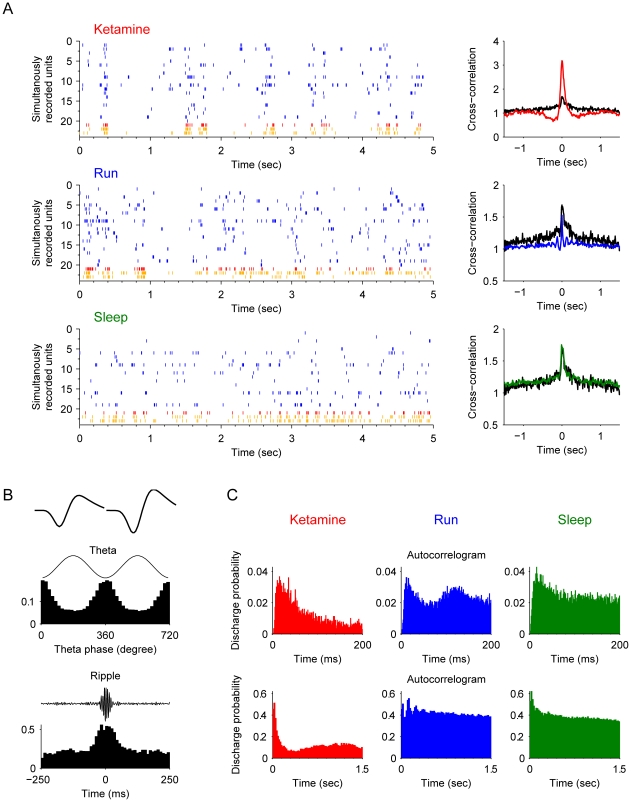
Properties of type-2 interneurons (putative *bistratified cells*). (**A**) Spike rasters of silmultanously recorded pyramidal cells (blue) and bistratified cells (an example unit plotted here in red and remaining units in orange) after ketamine injection, during running and slow-wave sleep are shown (left panels). The cross-correlation functions of the interneuron and pyramidal cell joint-activities are plotted on the right. The black line in each plot is the cross-correlation during the awake rest state. (**B**) The basic properties of this example basket cell. The most upper left panel shows the average spike waveforms. The middle left panel illustrates the spike timing of this unit is negatively coupled with theta oscillations (p<0.001, Rayleigth's test). The lower left panel shows the significantly elevated firing rate throughout the entire ripple epochs. (**C**) Autocorrelograms in different states: after ketamine injection, during running, and during sleep respectively. Autocorrelograms are shown in two time scales: 0–200 ms and 0–1.5 sec.

Type-2 interneurons, which have characteristics of *bistratified cells*, were also highly synchronized with the pyramidal cells during the ketamine-induced anesthesia which was different from running and sleep (Figure 5A and 5B). These bistratified cells, which are known to innervating str. radiatum and oriens, fire at the trough of the theta (Figure 5B) and with high frequency throughout the entire ripple episodes (Figure 5B). The electrophysiological difference between bistratified cells and basket cells are the mean theta phase of the firing spikes. Bistratified cells usually fire *at* the trough of the theta, while basket cells tend to fire *before* the trough of the theta oscillations. After ketamine injection, these bistratified cells oscillated together with pyramidal cells, similar to basket cells. Basket cells and bistratified cells together represented a large number of recorded interneurons (41 out of 99).

**Figure 6:**
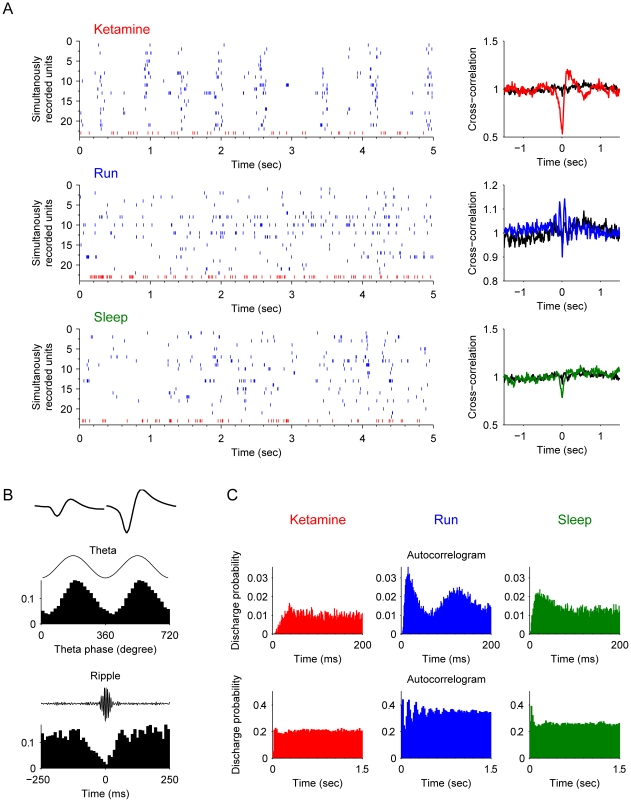
Properties of type-3 interneurons (putative *chandelier cell*, also termed as *axoaxonic cell*). (**A**) Spike rasters of chandelier interneurons (red) and simultaneously recorded pyramidal cells (blue) after ketamine injection, during running and slow-wave sleep are show (left panels). The cross-correlation functions of the interneuron and pyramidal cell joint-activities are plotted on the right. The black line is the cross-correlation during the awake rest state. The upper right panel shows this unit has negative correlation with pyramidal cell activities. (**B**) The properties of this example interneuron. The most upper left panel shows the average spike waveforms. The middle left panel illustrates the spike timing of this unit is strongly coupled with theta oscillations (p<0.001, Rayleigth's test). The lower left panel shows the firing rate significantly decreased during ripple epochs. (**C**) Autocorrelograms in different states: after ketamine injection, during running, and during sleep respectively. Autocorrelograms are shown in two time windows: 0–200 ms and 0–1.5 sec.

Type-3 interneurons belonged to another class of interneurons, namely, putative *chandelier cell* (or termed *Axo-axonic cells*) which exhibit slightly narrower waveforms and exhibited rhythmic property upon ketamine injection (Figure 6A). During theta oscillations these chandelier cells exhibited the highest firing around the peak of the theta (Figure 6B), but they became silent at the highest peak of the ripple episodes (Figure 6B). Unlike basket cells and bistratified cells, chandelier cells exhibited strong negative correlation with pyramidal cells after ketamine injection (Figure 6A). They were silent when pyramidal cells were active, and then became active when pyramidal cells were silent.

**Figure 7:**
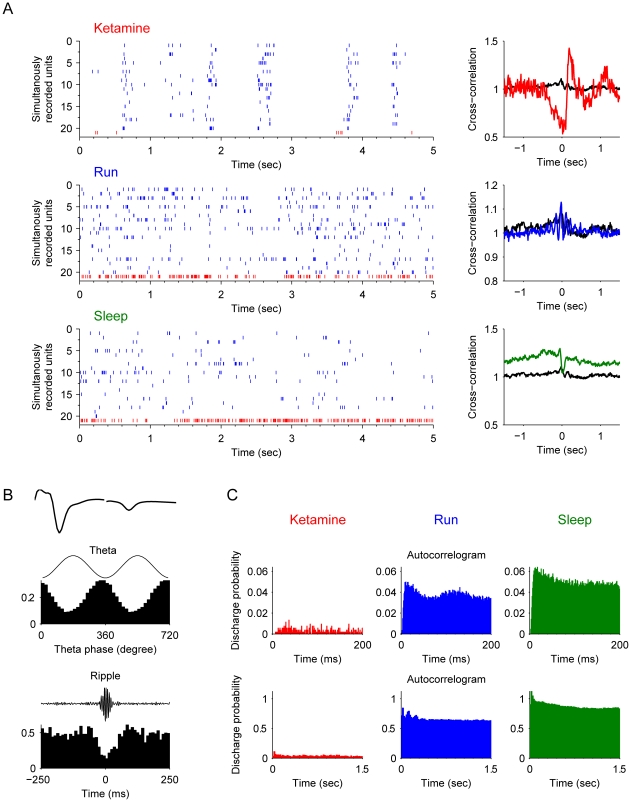
Properties of type-4 interneurons (putative O-LM cell). (**A**) Spike rasters of the putative O-LM cell (red) and simultaneously recorded pyramidal cells (blue) after ketamine injection, during running and slow-wave sleep are shown (left subpanels). The cross-correlation functions of the interneuron and pyramidal cell joint-activities are plotted on the right. The black line is the cross-correlation during the awake rest state. This unit has negative correlation with pyramidal cells at time 0 in the cross-correlation plot (upper right panel). (**B**) The basic properties of this O-LM cell. The most upper left panel shows the average spike waveforms. The middle left panel illustrates that this unit has strongly tendency to fire in the valleys of theta oscillations (p<0.001, Rayleigth's test). The lower left panel shows the firing rate is significantly decreased during ripple epochs. (**C**) Autocorrelograms in different states: after ketamine injection, during running, and during sleep respectively. Autocorrelograms are shown in two time scales: 0–200 ms and 0–1.5 sec.

Type-4 interneurons also exhibited the similar negative correlation with pyramidal cells during the ketamine-induced unconscious state in comparison to other cognitive states (Figure 7A). Type-4 interneurons fit the criteria of the putative *O-LM cells*. The O-LM cells fire at the trough of the theta episode, but became suppressed at the peak of the theta. Their firing was also significantly suppressed for the duration of ripple episodes (Figure 7B). One noticeable feature is that O-LM cells seemed to have much higher basal activity during sleep than during the unconscious state-induced by ketamine (Figure 7C).

**Figure 8:**
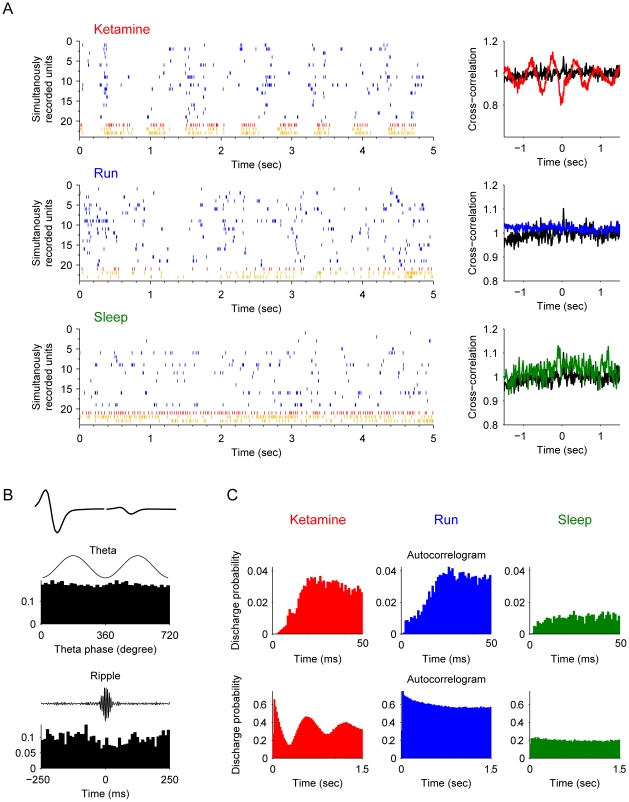
Properties of type-5 interneurons. (**A**) Spike rasters of silmultanously recorded pyramidal cells (blue) and type-5 interneuron (an example unit plotted is in red, and other type-5 interneurons in orange) after ketamine injection, during running and slow-wave sleep are shown (left subpanels). The cross-correlation functions of the interneuron and pyramidal cell joint-activities are plotted on the right. The black line is the cross-correlation during the awake rest state. (**B**) The basic properties of this type-5 unit. The most upper left panel shows the average spike waveforms. Firing probability histograms suggest that the neuronal firing timing showed slight correlation with theta oscillations but was not coupled to ripple oscillations (middle left and lower left panels). (**C**) Autocorrelograms in different states: after ketamine injection, during running, and during sleep respectively. Autocorrelograms are shown in two time scales: 0–50 ms and 0–1.5 sec. Note the slow oscillatory dynamics-induced by ketamine is evident in both cross-correlation plot (the top right corner plot in A) and the longer time scale autocorrelogram (bottom left corner plot in C).

Type-5 interneurons displayed slow oscillatory dynamics with an average frequency of 1.4 Hz after ketamine injection (Figure 8A). Some of type-5 interneurons had symmetric waveforms and some had asymmetric waveforms. The waveform of a representative unit is shown in the upper left panel of Figure 8B. Firing probability histograms suggest that the neuronal firing timing showed slight correlation with theta oscillations, but they were not coupled with ripple oscillations (see middle left and lower left panels of Figure 8B). Distinctively, autocorrelograms after ketamine injection show multiple peaks with approximately 0.7 sec intervals (see right panels of Figure 8C).

**Figure 9:**
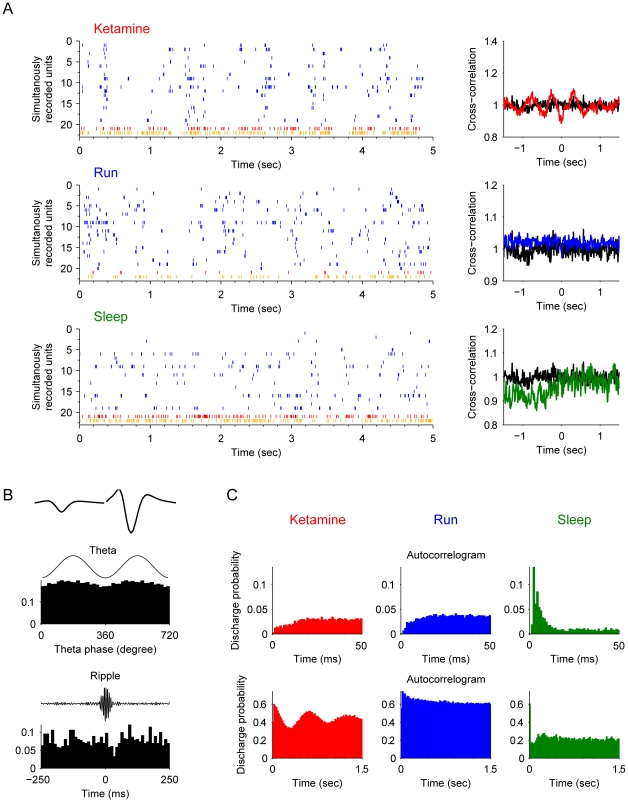
Properties of type-6 interneurons. (**A**) Spike rasters of silmultanously recorded pyramidal cells (blue) and type-6 interneurons (red unit is plotted for B and C) during ketamine anesthetic state, running, and slow-wave sleep are shown (left subpanels). Other two simultaneously recorded type-6 interneurons are in orange. The cross-correlation functions of the interneuron and pyramidal cell joint-activities are plotted on the right. The black line is the cross-correlation during the awake rest state. (**B**) The basic properties. The most upper left panel shows the average spike waveforms. Firing probability histograms during theta and ripples are shown in middle left and lower left panels respectively. (**C**) Autocorrelograms in different states: after ketamine injection, during running, and during sleep respectively. Autocorrelograms are shown in two time scales: 0–50 ms and 0–1.5 sec. Note the slow oscillatory dynamics are shown in both the cross-correlation plot and the larger time scale autocorrelogram after ketamine injection. This unit exhibits a burst firing pattern during sleep shown in the peak within 10 ms in the autocorrelogram.

Type-6 interneurons exhibited the similar ketamine-induced dynamics as those of type-5 interneurons (Figure 9A), but most of them had symmetric narrow waveforms with a valley flanked by two positive peaks (Figure 9B). Again, like type-5 interneurons, the firing of some type-6 cells was very weakly coupled to theta oscillations, but none to ripple oscillations (see middle left and lower left panels of Figure 9B). The difference between type-5 and type-6 interneurons is that the type-6 interneurons fired in sharp bursts during sleep, whereas type-5 did not (Figure 9C). But both type-5 and type-6 interneurons responded with rhythmic dynamics after ketamine injection. However, we also noted that some of the type-6 interneurons did not show such slow oscillatory patterns, which may indicate different types of cells within this subclass.

**Figure 10:**
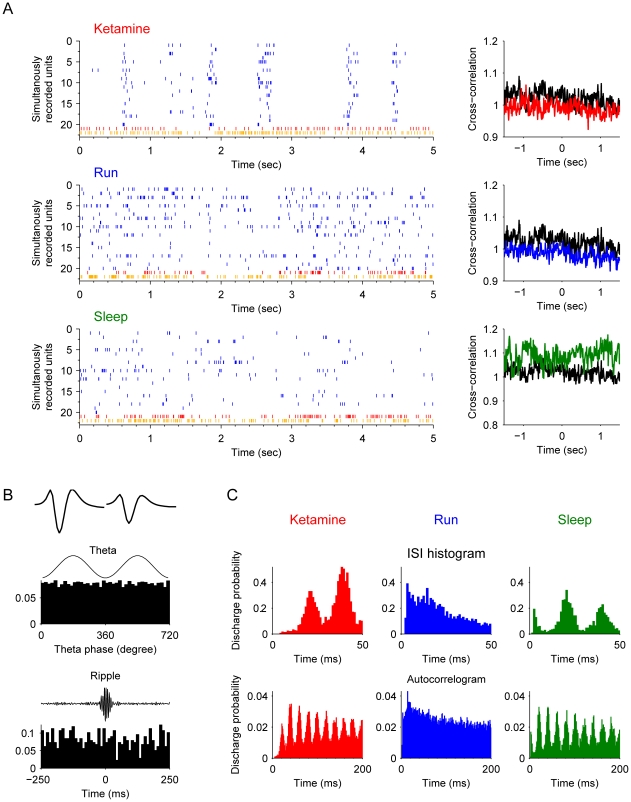
Properties of type-7 interneurons. (**A**) Spike rasters of silmultanously recorded pyramidal cells (in blue) and type-7 interneurons after ketamine injection, during running and slow-wave sleep are shown (left subpanels). The unit plotted for B and C is in red, and the second type-7 cell simultaneously recorded is in orange. The cross-correlation functions of the interneuron and pyramidal cell joint-activities are plotted on the right. The black line is the cross-correlation during the awake rest state. (**B**) The basic properties. The most upper left panel shows the average spike waveforms. Firing probability histograms suggest that the neuronal firing timing was not coupled to theta oscillations nor ripple oscillations (middle left and lower left panels). (**C**) ISI histograms and autocorrelograms in different states: after ketamine injection, during running, and during sleep respectively. Note the rhythmic peaks with 20 ms intervals in the sleep state and ketamine-induced anesthetic state.

Finally, we also identified another class of interneurons (type-7 interneurons) which exhibited very unique firing dynamics during the ketamine-induced unconscious state (Figure 10A). Type-7 interneurons had symmetric narrow waveforms with two positive peaks. The waveform of a representative unit is shown in the upper left panel of Figure 10B. Firing probability histograms suggest that the neuronal firing timing was coupled to neither theta oscillations, nor to ripple oscillations (see middle left and lower left panels of Figure 10B). Distinctively, ISI histograms and autocorrelograms after ketamine injection show multiple peaks with about 20 ms intervals (see right panels of Figure 10C), with some neurons have the first peak about 4–6 ms. This indicates that these neurons fire regularly with 20 ms or multiple of 20 ms intervals (∼50 Hz). These cells had the similar characteristic 50 Hz firing rate during slow-wave sleep (Figure 10C). On the other hand, ISI histogram and autocorrelogram from the running or awake states did not show this feature (middle panels of Figure 10C).

**Figure 11:**
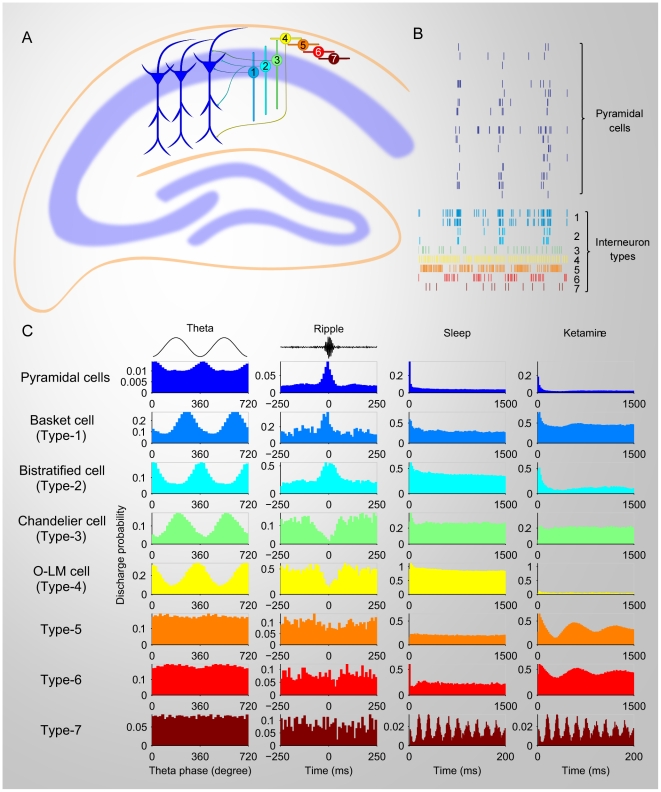
Summary of distinct firing dynamics of pyramidal cells and 7 different types of interneurons in CA1. (**A**) The chart illustrates the anatomical positions of pyramidal cells and interneurons. Type-1 (basket cell), type-2 (bistratified cell) and type-3 (chandelier cell) are positioned in the pyramidal cell body layer in the hippocampus CA1 subregion. Type-4 (O-LM cell), Type-5, type-6, and type-7 interneurons located in the str. oriens. (**B**) Firing temporal rhythms of pyramidal cells and seven distinct types of interneurons under ketamine anesthesia. (**C**) Distinct profiles of distinct CA1 cells in relationship with theta oscillations (the first columns of plots from left), ripples (second colomun), and their autocorrelograms during sleep (third column) and ketamine-induced anesthesia (right column).

Type-1 (basket cell), type-2 (bistratified cell) and type-3 (chandelier cell) are positioned in the pyramidal cell body layer in the hippocampus CA1 subregion. As expected, these neurons were usually simultaneously recorded together with pyramidal cells. On the other hand, our recording suggests that O-LM cells, type-5, type-6, and type-7 interneurons were recorded slightly above the pyramidal cell body layer, namely, in the str. Oriens. (see the illustrative diagram in Figure 11). Distinct classes of neurons showed a sophisticated temporal dynamics during the anesthesia state induced by ketamine.

## Discussion

Traditional view is that dissociative anesthesia produced by ketamine is a result of reduced activation in the thalamocortical structures and increased activity in the limbic system and hippocampus [1]. Our ensemble recording technique reveals that ketamine induced synchronized firings among hippocampal CA1 populations. This occurred despite the fact that the average firing rates of pyramidal cells and interneurons were slightly reduced.

It is known that pyramidal cells and diverse interneurons compose the intricate hippocampal circuits and are involved in various firing patterns in the hippocampus during spatial running. Important questions remain as to whether and how various interneurons interact with pyramidal cells and contribute to the synchronous firing of CA1 network-induced by ketamine. Much of current knowledge has been obtained from studies of *in-vitro* brain slices. Little is known about its detailed action on dynamic patterns of hippocampal cells *in vivo*. And the network level study is limited because of the small number of simultaneously recorded neurons. By taking the advantage of our large-scale *in-vivo* neural recording technique, we are allowed to monitor many pyramidal cells and interneurons at the same time. Although it is not as accurate in distinguishing different neuron types as the studies on brain slices, which use different molecular markers, but we can classify the recorded interneurons into at least seven major types, including known and unknown types, based on their distinct firing patterns and compare with the *in vitro* results. Type-1 and type-2 interneurons were CA1 basket cells and bistratified cells according the characteristics of these cells [34], [35], [46] were made of nearly half of recorded interneurons. These cells innervate pyramidal cell somas and dendrites. After ketamine injection, they were in tight synchronization with pyramidal cells.

Type-3 and type-4 interneurons are putative chandelier cells and O-LM cells, respectively. These interneurons tended to fire during the period when pyramidal cells were silent. Cross-correlation analyses confirmed this negative dynamic correlation with pyramidal cells. The above four types of interneurons all exhibited dynamic relationships with the theta and ripple episodes which provided the characteristic classifications to their putative identities [34], [35], [46].

As expected, basket cells, bistratified cells, and chandelier cells were usually simultaneously recorded together with pyramidal cells. On the other hand, our recording suggests that O-LM cells, type-5, type-6, and type-7 interneurons were recorded slightly above the pyramidal cell body layer, namely, in the str. Oriens (Figure 11). While the firing of these interneurons was clearly altered by ketamine, they were either weakly or not coupled to theta oscillations. They also did not fire in temporal relation to ripple episodes. At the moment, we could not assign type-5, type-6, and type-7 interneurons to previously described interneuron types. There are trilaminar cells, back-projection cells, hippocampo-septal cells in the str. oriens [34], [35], [46]. It is worthy to note that the type-7 interneuron displays a unique distribution of inter-spike intervals, resulting in multiple peaks at multiples of 20 ms in both ISI histogram and autocorrelogram. This characteristic firing is present during the slow-wave sleep and ketamine-induced unconscious states, but not during the awake states. Because the interneurons with their cell bodies located in the str. oriens (such as trilaminar cells, back-projection cells, hippocampo-septal cells region) tend to be long-range GABAergic projections[34], [35], [46], the strong rhythmic firings of type-5, type-6 and type-7 interneurons may play an important role in coordinating oscillatory timing between the hippocampus and the cortical areas as well as the septum. Because the type-7 interneurons exhibited nearly identical discharge probability during two unconscious states (slow wave sleep and ketamine-induced anesthesia), it is of special interest to investigate the cellular and molecular identities of the type-7 cells.

While ketamine acts primarily by as an uncompetitive NMDA receptor antagonist, ketamine can act on other receptors or channels including GABAa receptor, nicotinic and muscarinic Ach receptors, etc [3] which also vary among different cell types. It is possible that the 50 Hz rhythmic firing of type-7 interneurons induced by ketamine or sleep might be the property of the trilaminar cells since they were reported to contain high amount of muscarinc Ach receptor (M2), and moreover, fired at 50 Hz spontaneously[34], [47], [48]. Given the role of the Ach in regulating attention and consciousness [49], it is of importance to test whether type-7 interneurons would respond to cholinergic agonists or antagonists in future experiments.

In future, it will also be important to combine both pharmacology and genetic methods to understand the contribution of NMDA receptors on distinct cell types to the above observed dynamics as well as to other oscillatory properties and patterns. It will also be of great interest to investigate how ketamine at a subanesthetic dose alters network dynamics underlying various encoding patterns of various types of hippocampal cells, leading to abnormal pattern generations with respect to the possible hallucination state.

In summary, our present study has examined neuronal activity patterns in hippocampal CA1 networks during the dissociative anesthetic state-induced by ketamine and compared with other natural conscious and unconscious state such as slow wave sleep. Our analyses reveal that ketamine induces not only the rhythmic firing of pyramidal cells but also various distinct firing patterns in many types of CA1 interneurons. In other words, the emergent coordinated firing patterns in CA1 pyramidal cells are tightly accompanied by an equally sophisticated temporal dynamics of various distinct classes of interneurons, thereby producing characteristic oscillatory dynamics in CA1 circuits. It is conceivable that systematic characterizations of these abnormal network dynamics induced by ketamine may eventually lead to better understanding of how unconscious states are produced.

## Materials and Methods

### Ethics Statement

All animal work described in the study were carried out in accordance with the guidelines laid down by the National Institutes of Health in the US regarding the care and use of animals for experimental procedures, and was approved by the Institutional Animal Care and Use Committee of the Medical College of Georgia (Approval AUP number: BR07-11-001).

### Animal surgery and *in vivo* recording

We employed 96-channel recording arrays to record from hippocampal CA1 region from freely behaving mice, same as previously described [37], [39]. The 96-channel recording electrode consists of two independently movable bundles of 48 stereotrodes (48 channels on each side of the hippocampi). Each stereotrode was constructed by twisting a folded piece of two wires (STABLOHM 675, H-FORMVAR, 25 µm, California Fine Wire), and spaced about 150 µm. Male wild-type B6BCA/J mice were handled for several days prior to surgery to minimize the potential stress of human interaction. On the day of surgery, the mouse was anesthetized with an i.p. injection of 60 mg/kg ketamine (Bedford Laboratories, Bedford, OH) and 0.5 mg/kg Domitor (Pfizer Animal Health, New York, NY). The electrode bundles were positioned above the bilateral dorsal hippocampi (2.0 mm lateral to the bregma and 2.3 posterior to the bregma on the both right and left sides). The mouse was then awoken with an injection of 2.5 mg/kg Antisedan. After surgery, the mouse was allowed to recover for three to five days. Then the electrode bundles were advanced slowly toward the hippocampal CA1 region, in daily increments of about 0.07 mm until the tips of the electrodes had reached the CA1, as deduced from an assessment of high-frequency ripples, field potential, and neuronal activity patterns [37], [39].

We subsequently recorded the ensemble activity of a large number of individual neurons during free movement, consisted of at least 20 minutes of sleep and awake. Then the animal was injected with 100 mg/kg ketamine in saline via i.p. After injection, the mice lost the righting reflex in 2–3 minutes, and started to recover half to one hour later. The recording was continued until the mouse had recovered from the ketamine injection.

At the end of the experiments, the mice were anesthetized and a small amount of current was applied to the recording electrodes in order to mark the positions of the stereotrode bundles. The actual electrode positions were confirmed by histological Nissl staining using 1% cresyl echt violet. The stability of the *in vivo* recordings was judged by waveforms at the beginning, during, and after the experiments. All animals received normal light-dark cycles and were not subjected to food and water deprivation or restrictions. Five adult, wild-type male mice (3–8 months old) were used for this experiment.

### Data processing and spike sorting

The neuronal activity was recorded by a MAP system (Plexon Inc., Dallas, TX). Extracellular action potentials and local field potentials data were recorded simultaneously and digitized at 40 kHz and 1 kHz respectively. The recorded spike activities from CA1 neurons were processed in a manner as described previously [39]. First, the spike waveforms and their associated time stamps for each of channels were filed as Plexon system format (*.plx). Before spike sorting, the artifact waveforms were removed and the spike waveform minima were aligned using the Offline Sorter 2.8 software (http://www.plexon.com, Dallas, TX). The aligned data were then written in the Neuralynx system format (*.nst). Finally, spike sorting was carried out by using an auto-clustering method (KlustaKwik 1.7) followed by manual cutting and merging in MClust 3.5 program (http://redishlab.neuroscience.umn.edu/MClust/MClust.html) to identify and isolate different spiking units. Only units with clear boundaries and less than 0.5% of spike intervals within a 1 ms refractory period are included in the present analysis. In general, putative pyramidal cells had wider waveforms (>300 µsec) and lower average firing rates (<5 Hz), whereas putative interneurons had relatively narrower waveforms (<250 µsec) and higher firing rates (>5 Hz). In addition, the discharge dynamics of putative pyramidal cells and putative interneurons were also characteristically different. Pyramidal cells are known to fire complex-spike bursts with 3–10 ms inter-spike intervals occasionally. Consequently, the autocorrelogram of pyramidal cells typically shows a characteristic peak at 3–5 ms, followed by a rapid exponential decay, whereas putative interneurons exhibited a later peak and a much slower decay. Further analysis is based on the sorted data.

### Field potential analysis

First, the recorded local field potentials (LFP) were processed by the FPAlign (a utility program provided by Plexon Inc.: http://www.plexoninc.com/support/softwaredownloads.html) to correct the filter induced phase delays. LFP channels recorded in pyramidal cell layer were selected by judging the maximum yield of ripple during animal slow-wave sleep (SWS), and comparing the coherence in the theta-frequency and gamma-frequency band [50]. Further analyses were carried out with these LFP data off-line by custom-written MATLAB (Mathworks) programs.

For extraction of ripple events, the LFP data was digitally band-pass filtered (100―250 Hz) by a hamming window based FIR filter with order of 30. The root mean square power of the filtered signal was calculated by sliding 10 ms window every one millisecond and averaged across electrodes. A threshold was set to 5 standard deviations (SDs) above the mean power to detect ripples. The beginning and the end of oscillatory epochs were marked at points with the power reduced to 2 SD above background mean. Periods of 20 ms or more were designated ripple episodes. The minimum point during each episode was regarded as the reference to calculate the peri-event histogram for each individual unit. If there are at least 3 bins during −30 to 30 ms (bin size 10 ms) greater than 2 SD above the mean (the −500 to −100 ms and 100 to 500 ms epochs were used as baseline), then this unit was considered having significant elevated firing rate during ripple episodes.

For detection of theta epochs, the theta/delta power ratio was calculated in a 2 s window. More than consecutive 15 s periods in which the ratio was greater than 4 were identified, then the beginnings and ends of epochs were judged manually. To calculate the phase relationship between unit activity and theta oscillation, the LFP data was first digitally band-bass filtered (4–12 Hz). The troughs for each cycles were identified and were assigned instantaneous phase of 0 radians. The phase value of each spike was obtained by linear interpolation. Then the histogram was calculated with 20 degree bin size, and a Rayleigh's test was performed to determine the significance of phase modulation of theta oscillations.

### Cross-correlation analysis

Spike activities of all pyramidal cells were pooled together to reconstruct the joint-activity data; then the data was converted to a spike occurrence sequence with a 10 ms bin. Cross-correlation function was performed between the occurrence sequences data of joint pyramidal cell activities and the occurrence sequences data from individual interneuron from different neuron classes. Auto-correlation function of pyramidal cells was performed within the joint-activities of pyramidal cells. The result was normalized by the period time and frequencies of the joint-activity of both reference class and test unit or test class. So if the firing time series of two classes are independent, the normalized cross-correlogram will be around 1; greater than 1 indicates a positive correlation; contrarily, less than 1 represents a negative correlation.
